# Modulation of Physical Activity to Optimize Pain Sensation following an Intra-Articular Corticosteroid Injection in Patients with Knee Osteoarthritis

**DOI:** 10.1155/2014/209165

**Published:** 2014-11-16

**Authors:** Yoann Dessery, Étienne L. Belzile, Sylvie Turmel, Jean Doré, Binta Diallo, Philippe Corbeil

**Affiliations:** ^1^Département de Kinésiologie, Faculté de Médecine, Université Laval, 2300 rue de la Terrasse, Quebec City, QC, Canada G1V 0A6; ^2^Unité de Recherche sur le Vieillissement, Centre de Recherche FRSQ du CHA Universitaire de Québec, 1050 Chemin Sainte-Foy, Quebec City, QC, Canada G1S 4L8; ^3^Division de Chirurgie Orthopédique, CHUQ, 11 Côte du Palais, Quebec City, QC, Canada G1R 2J6; ^4^Département de Chirurgie, Faculté de Médecine, Université Laval, 1050 avenue de la Médecine, Quebec City, QC, Canada G1V 0A6

## Abstract

*Background*. Intra-articular corticosteroid injection is often used to relieve pain caused by knee osteoarthritis. This study aims to assess the impact after an intra-articular corticosteroid injection treatment on objective and subjective measurement of physical function in knee osteoarthritis patients.* Methods*. Fourteen patients with unilateral knee osteoarthritis participated in this open-label uncontrolled trial. The intra-articular corticosteroid injection was given at the end of the second week. Physical activity was objectively measured by an accelerometer worn by the participants for eight weeks. Symptoms, quality of life and spatiotemporal parameters of gait were assessed every two weeks.* Results*. From the injection until six weeks later, pain and stiffness were reduced by approximately 60%. Patients' daily physical activity time was significantly improved after injection: participation in light and moderate physical activities increased during four and two weeks, respectively.* Conclusions*. The beneficial effects after the intra-articular corticosteroid injection are visible in the duration and intensity of the knee osteoarthritis patients' daily physical activity. However, these effects declined gradually two weeks after injection. Modulating the intensity and duration of physical activity would allow patients to optimize pain sensation over a longer period following an intra-articular corticosteroid injection.* Trial Registration*. This trial was registered with ClinicalTrials: NCT02049879.

## 1. Background

Osteoarthritis is the most common form of arthritis in the world. It is a chronic, degenerative, and noninflammatory disease, which predominantly affects the fingers and load-bearing joints such as hips and knees [1]. In the United States, 33.6% of adults aged 65 and older had osteoarthritis in 2005 [2] and 12.1% of those aged 60 and older had symptomatic radiographic knee osteoarthritis (KOA) [3]. KOA is one of the leading causes of disability and results in reduced activity in people over 50-year old. Three types of treatment are recommended: nonpharmacological, pharmacological, and surgical [4]. The aim of any treatment in KOA is to decrease pain, maintain or improve articular mobility, and increase physical function.

From the 1950s to the present, rheumatologists have used intra-articular corticosteroid (IAC) injections to reduce joint pain and increase joint mobility. According to Ayhan et al., IAC should be considered an adjunct to core treatment for the relief of moderate to severe pain in people with osteoarthritis and as the last nonoperative modality, if the other conservative treatment modalities are ineffective [5]. Thus, in the United States, 95% of them employ this pharmacological treatment at least “sometimes” and 53% use it “frequently” [6]. Unlike oral therapy, a local injection avoids serious adverse effects such as muscle weakness, gastrointestinal and renal toxicity, and peptic ulceration [4, 7]. Osteoarthritis Research International (OARSI) gave IAC injection a recommendation strength of 78% [4]. IAC injections provide short term reduction in osteoarthritis pain, usually felt one week after the injection [8]. However, this relief lasts for only two to three weeks [8, 9]. Moreover, no significant difference is found between IAC injections and placebos regarding physical function or patient global assessment as measured by a visual analog scale [8].

Quantification of functional capacity to accomplish daily physical activities is an important variable affecting the quality of life (QoL) of individuals with reduced mobility [10]. Indeed, physical inactivity is the fourth leading risk factor for global mortality [11]. Several objective and subjective measuring tools are currently used to evaluate the effects of treatment. Thus, self-administered questionnaires (e.g., WOMAC and MOS-SF-36) [12, 13] are commonly used to assess physical capacity, QoL, or pain. Although these questionnaires are widely applied, they are sensitive to patients' memory or errors, so their validity and the usefulness of the data could be corrupted [14, 15]. On the other hand, physical performance tests such as the Tinetti mobility test and the Timed Up and Go test can give more objective results regarding balance and gait performance [16, 17]. However, scores obtained with these tests depend on the assessors' judgment and experience [18]. Furthermore, these tests evaluate a subject's functional capacities at a specific point in time and in a clinical setting, so assessments are contingent on patients' condition when the tests are administered.

Over the last few years, new tools have allowed for continuous recording of the daily physical activity parameters (e.g., steps and calories) in an objective way and in a real-life environment [18–20]. Uniaxial accelerometers/pedometers have been used to evaluate mobility in people with orthopedic [18, 19, 21], neurological [22], pulmonary [23], and cardiac disorders [24]. Brandes et al. [18] mentioned that walking tests and questionnaires are not able to evaluate the mobility level of people suffering from knee or hip osteoarthritis in their living environments as effectively as an activity monitor pedometer.

The objective of this study was to objectively measure daily physical activity and spatiotemporal gait pattern, as well as improvements in self-reported symptoms and quality of live before and six weeks after an IAC injection in patients suffering from KOA. We hypothesized that light and moderate physical activity, as well as gait velocity, would increase during the first two weeks after the injection and gradually decline in the following weeks.

## 2. Methods

This study is an open-label trial of intra-articular corticosteroid injection with KOA patients. The trial was registered at ClinicalTrials.gov (Identifier: NCT02049879) after enrolment of participants due to communication problems. The authors confirm that all ongoing and related trials for this intervention are registered. No control group was included in this study. The protocol for this trial and supporting CONSORT checklist are available as supporting information; see Checklist and Protocol.

### 2.1. Participants

**Figure 1:**
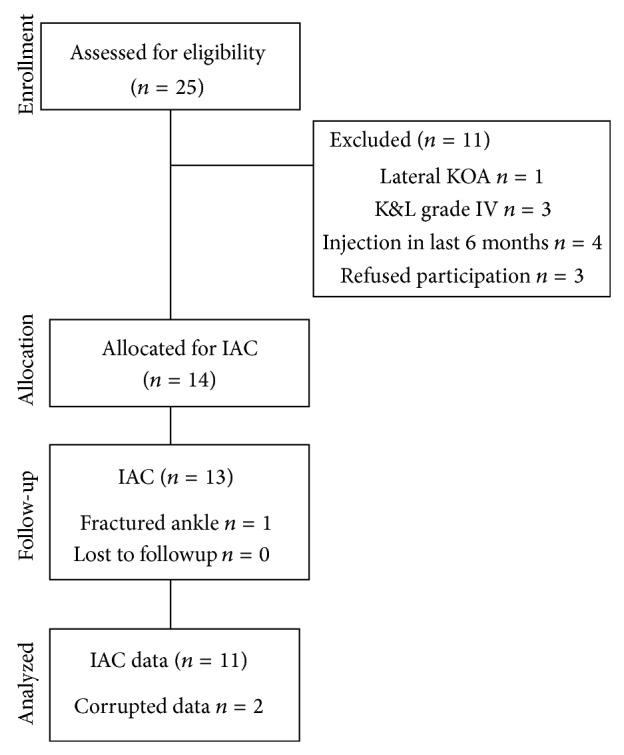
Flow diagram of the study (according to CONSORT statement). Diagram illustrates recruitment of patients suffering from KOA, reasons for exclusion, and the treatment received, including 6-week follow-up.

Between November 11, 2008, and July 5, 2012, 14 participants (7 women and 7 men) presenting medial KOA symptoms were recruited to participate in our study (Figure 1). The study period lasted for a further 3 months after enrolment of the last participant. Their mean (SD, range) age, weight, height, and BMI were 62.5 years (8.6 years, 50–84 years), 80.1 kg (15.4 kg, 46.3–103 kg), 1.67 m (0.12 m, 1.48–1.85 m), and 28.8 kg/m^2^ (4.3 kg/m^2^, 20.6–37.8 kg/m^2^).

A convenience sampling method was applied. Participants were referred by an orthopedic surgeon, who was informed of the study's inclusion and exclusion criteria. KOA was diagnosed according to the American College of Rheumatology's clinical and radiological criteria. Participants were included if they were more than 50-year old, had been diagnosed with isolated medial compartment KOA (Kellgren & Lawrence Grades 1 to 3), and had no history of intra-articular injection during the last six months. They were excluded if they had isolated femoropatellar osteoarthritis, rheumatoid arthritis, knee instability, spinal stenosis, lower limb fracture over the last year, or surgery in the last three months. Ethics approval for the study was obtained from Université Laval ethics committee. Participants were fully informed about the nature, goal, procedures, and risks of the study and gave their informed consent in writing.

### 2.2. Experimental Procedure

For each enrolled patient, participation lasted eight weeks and involved five clinical encounters. At the initial patient interview, an activity monitor pedometer (Kenz Lifecorder, LCPLUS;* Suzuken Co. Ltd., Nagoya, Japan*) was handed over. Uniaxial accelerometers are commonly used to evaluate free-living activity and energy expenditure [25, 26]. The LCPLUS activity monitor was worn on the belt from the time the patient got out of bed in the morning until he or she went to bed at night, every day of the week, for eight weeks. At the second meeting, an evaluation was done to assess the patient's baseline disease state, and the patient was given an intra-articular corticosteroid injection in a sitting position. Injection was carried out by two different orthopedic specialists and all injections contained a solution of triamcinolone 40 mg (Kenalog) mixed with 3 cc of 2% xylocaine without epinephrine. After the IAC injection, standardized recommendations for all patients suggested three days' rest. The next three visits were scheduled at weeks 2, 4, and 6 after injection. At each clinical meeting, patients were asked to complete two questionnaires (WOMAC and MOS-SF-36) and to undergo gait analysis on an instrumented walkway. For the gait analysis, participants were instructed to walk across a 14-foot sensor map (GAITRite electronic walkway,* CIR Systems, Havertown, PA*) at their preferred gait speed. This was done ten times. The sensor map was positioned so as to allow each participant to reach a steady-state walk before stepping onto it. Patients' spatiotemporal gait parameters were computed with GAITRite software.

### 2.3. Outcome Measurement

The French version of the Western Ontario and McMaster Universities Osteoarthritis Index (WOMAC), with a visual analog scale for each question, was used to determine the impact and severity of osteoarthritis in the patients suffering from KOA. The scale is divided into three subscales, each including several items: pain (5 items), stiffness (2 items), and physical function (17 items). Higher scores indicate greater disease severity. Also, the participants' QoL was assessed with the Canadian (French) version of the Medical Outcomes Study 36-Item Short Form (MOS SF-36). This questionnaire measures eight domains of QoL with a Likert-style scale: physical functioning, limitations due to physical health, bodily pain, general health, vitality, social functioning, limitations due to emotional health, and mental health. It is a common generic index used in many studies. And, unlike the WOMAC, higher scores indicate better condition. These two questionnaires give results on a 100-point scale.

Among spatial-temporal gait parameters, step velocity, cycle duration, cadence, stride length, and walking base (step width) were averaged over the ten walk trials.

The number of kilometers covered by the participants each day, a step count per minute, estimated energy expenditure, and a record of the physical activity intensity at 4 s intervals were provided by the activity monitor. Activity time is defined as the daily activity duration with a level of intensity superior to 0.5 Metabolic Equivalent of Task (MET; 1 MET is equal to the energy produced per unit surface area of an average person seated at rest).

### 2.4. Statistical Analyses

Power analysis has been used to determine the number of required patients. Knee pain, gait velocity, and walking distance were defined as the primary parameters. Knee pain power analysis was based on Skwara et al. and Shrader et al.'s studies [27, 28] while gait velocity and walking distance power analyses were conducted from partial eta-squared of our preliminary results (*N* = 8). We found partial eta-squared equal to 0.197 and 0.447 for gait velocity and walking distance, respectively. Based on these three power analyses, a maximum sample size of 10 patients was required for *α* = 0.05 and power = 0.80. Therefore, we included 14 patients in our study.

A one-way repeated measures ANOVA was used to assess the effects after a treatment over six weeks (baseline, after 2 weeks, after 4 weeks, and after 6 weeks) on each dependent variable (Statistica 7,* StatSoft, Inc., Tulsa, OK*). The sphericity assumption was tested using the Mauchly's test. When sphericity was violated, Greenhouse-Geisser correction was applied to adjust the degrees of freedom for the averaged tests of significance. The estimate of effect size (ES), that is, partial eta-squared estimate, when comparing conditions at each time point, was calculated following the scale proposed by Cohen [29]: small (ES > 0.2), moderate (ES > 0.5), or large (ES > 0.8). When necessary, post hoc analyses were performed using Tukey's HSD test. Statistical significance was set at *P* < 0.05.

## 3. Results

One participant was excluded from the study due to a broken ankle between the third and the fourth visit. All the other participants completed the whole experimental protocol. A technical problem with two pedometers led to data loss for two participants. Thus, the statistical analyses were carried out on gait parameters and measures obtained from the questionnaires for 13 patients and on daily physical activity measures for 11 patients. No adverse side effects of injection were reported.

### 3.1. Questionnaires

**Figure 2:**
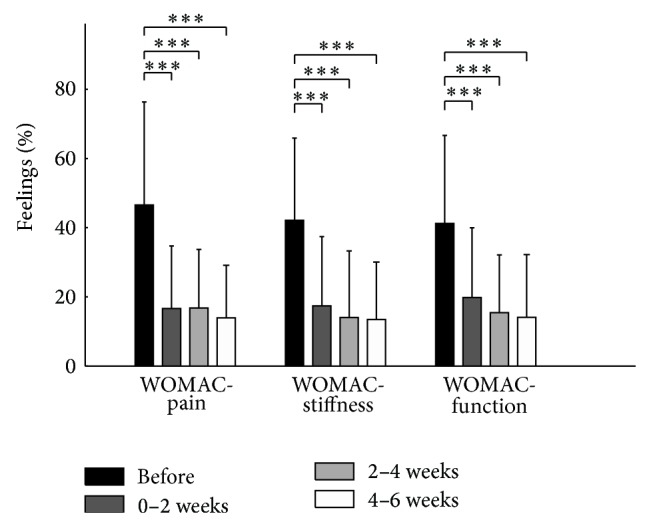
Answers to the questionnaires divided into different domains and collected before injection and every two weeks after injection for six weeks. ^***^ *P* < 0.001.

Pain, stiffness, and function of the affected limb were reduced by 66%, 64%, and 60%, respectively, from the second to the sixth week after the IAC injection when compared to baseline value (ES = 0.61, 0.50, and 0.54, resp.; powers = 1.0; Figure 2). No difference was observed between postinjection values (2nd versus 4th week: *P* > 0.79; 2nd versus 6th week: *P* > 0.62; 4th versus 6th week: *P* > 0.94). Change in QoL was identified for four of the eight items of the SF-36 (Table 1). With regard to the baseline, the IAC injection improved bodily pain and social functioning from the second to the sixth week after injection and vitality (energy/fatigue) and limitation due to physical health during the four last weeks (Table 1).

### 3.2. Spatiotemporal Gait Parameters

Gait analysis demonstrated that the KOA patients' gait pattern was transiently but significantly affected (Table 2). After two weeks, patients had significantly increased their self-selected walking velocity (*P* = 0.012), and this lasted until the sixth week after the injection (baseline versus 4th week: *P* = 0.032; baseline versus 6th week: *P* = 0.016). When compared to the baseline, cadence increased after the injection (*P* < 0.05), stride length was increased only after two weeks (*P* = 0.015), and walking base was not affected by the corticosteroid treatment (*P* = 0.13). Gait cycle duration time was reduced after the fourth (*P* = 0.022) and sixth weeks (*P* = 0.030).

### 3.3. Daily Physical Activity Measure

**Figure 3:**
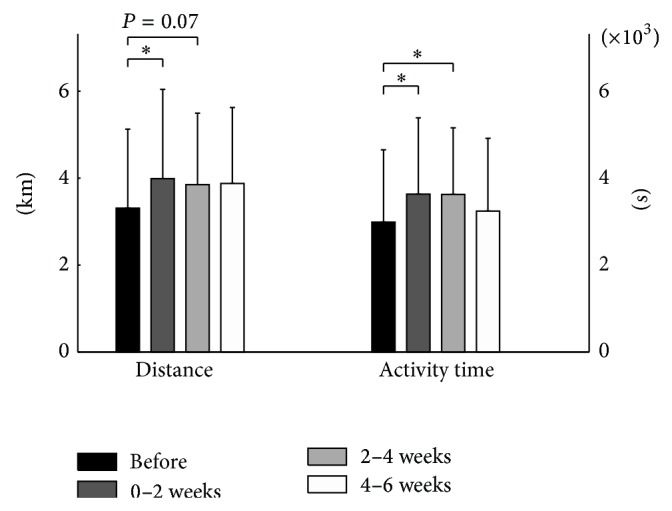
Mean daily distance covered and activity time during the two-week periods from two weeks before the injection to six weeks after the injection. ^*^ *P* < 0.05.

**Figure 4:**
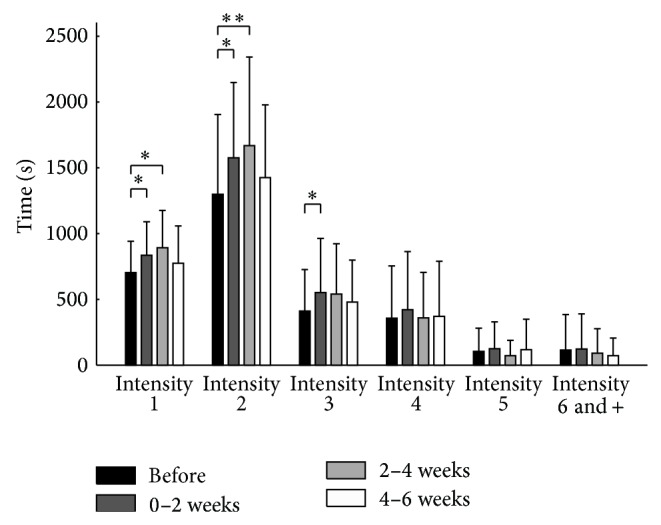
Mean daily intensity level (intensity 1 = 1 MET, intensity 2 = 2 METs, and so on) of physical activity during the two-week periods from two weeks before the injection to six weeks after the injection. ^*^ *P* < 0.05; ^**^ *P* < 0.01.

When compared to the baseline, daily walking distance and activity time increased by 20% (*P* = 0.015) and 21% (*P* = 0.012), respectively, in the first two weeks and by 13% (*P* = 0.066) and 17% (*P* = 0.014) in the following two weeks compared to the baseline (walking distance: ES = 0.31 and power = 0.84; activity time: ES = 0.34 and power = 0.89 for activity time; Figure 3). Time spent performing light (1 or 2 METs) and moderate (3 METs) physical activities was improved two weeks following injection (Figure 4). Improvement in the subsequent weeks was only observed for the light intensity level of activities.

## 4. Discussion

This study is the first to assess the effects after a IAC injection on daily physical activity among knee osteoarthritis patients. It is well known that IAC injection leads to pain relief, so we sought to determine whether this relief had a positive effect on participation in physical activity during the day and on gait pattern. In spite of a small sample and the absence of control group, the results of this study showed an improved daily distance traveled and an increased duration of activities performed at light and moderate intensity levels. However, the effect after the IAC injection on daily physical activity did not last as long as the symptom relief.

Many studies have shown that IAC injection is an effective treatment for reducing pain; its effects set in quickly, but they are short-lived; that is, they last less than three weeks [30–32]. Likewise, our results showed a quick decrease in pain by 50% two weeks after injection, but contrary to other studies [30–32], this pain relief lasted for six weeks after the injection. Furthermore, whereas other authors reported small or no change in the stiffness and function subscales [33–35], in our study, these two variables were greatly improved in the six weeks after injection (~50%). These discrepancies may be explained by the questionnaires used to evaluate stiffness and function (e.g., Lequesne Index and Health Assessment Questionnaire). Raynauld et al. [35] used the WOMAC questionnaire and found no difference between the scores obtained before and after treatment. However, in their study, the questionnaire was filled out three months after the injection whereas injection has only short-term effects. The effects on function and stiffness may therefore last between six and twelve weeks after IAC. Following these changes, patients' QoL was also improved. The KOA patients mentioned that they felt less constrained physically and had more vitality, thus improving their social lives.

In KOA, biomechanical alterations of the lower limbs, such as misalignment of the limb, are accompanied by gait pattern alterations [36, 37]. Some authors have reported a decline from 7% to 16% in the self-selected speed due to the reduction of the stride length and augmentation of the cycle duration in KOA patients [36–38]. IAC injections lead to an increase in the hip, knee, and ankle adduction moment 15 minutes afterward but have no effects on these variables and vertical ground reaction force one and eight weeks later [27, 28]. Though the effect of IAC injections on gait velocity is visible 15 minutes after the injection [28], this effect is no longer after one week and after eight weeks. The KOA patients in our study experienced significant improvement in overall spatiotemporal parameters and so walked with a pattern closer to the normal gait pattern until at least six weeks after the IAC injection. However, one study reported that the combination of pain relief and an increased gait velocity was associated with an increase in maximum knee adduction moment, that is, medial knee loading [28]. An increase in loading of the medial compartment of the knee is a well-known biomechanical risk factor for progression of KOA [39, 40]. Thus, one could debate, as other authors have, whether IAC injection treatment alone should be recommended to KOA patients as it could increase joint wear through medial knee loading. Other treatment modalities permitting modifications of the alignment of the lower limb, such as knee unloading braces or lateral wedged insoles, should probably accompany a pharmacological treatment aiming to relieve pain [41] and decrease medial knee loading.

Daily activities are categorized by intensity: light (<3 METs), moderate (3–6 METs), or vigorous (>6 METs) [42]. The World Health Organization [11] recommends that people engage in a minimum of 150 minutes per week of moderate-intensity physical activity to improve or maintain their general physical health, maintain mental health, and reduce the risk of noncommunicable disease. The Exercise and Physical Activity Conference (EPAC) published specific recommendations for KOA patients, advising them to accumulate 30 minutes of at least a moderate-intensity physical activity on at least three days a week [43]. Moreover, physical activity is also recommended to manage the pain caused by KOA [4], decrease the stiffness of osteoarthritic joints, and maintain or reduce body weight. However, a recent study has shown that 62% of KOA patients failed to follow the EPAC recommendations [44]. In the present study, following IAC injection, an improvement (up to 20%) in patients' light and moderate activity time and daily walking distance was observed during the first two weeks. The gradual decline in moderate-intensity physical activity two weeks after the IAC injection, followed by a decline in walking distance and overall activity time after the fourth week, may explain why pain relief after injection was extended beyond the six-week mark. These adaptations (i.e., adjusting the intensity and duration of physical activity each day) would allow patients to optimize pain sensations over a longer period following the injection. It is interesting to note that, from a cross-cutting perspective, preferential gait speed six weeks after injection remained higher than the baseline measurement. Clinical measurements taken at a specific point in time do not necessarily provide information about the degree of stress experienced in physical functioning in day-to-day situations. This finding indirectly confirms the value and necessity of continually assessing physical function objectively and in a real-life environment.

Our study has some limitations. First, we chose to expose our sample of patients to corticosteroid injection only and we did not use a control placebo treatment. Thus, significant differences found in our results could be due to the placebo effects of an intra-articular injection. Another study [8] has shown that the placebo effect is smaller than the IAC treatment effect for pain in KOA. Moreover, Jones and Doherty [30] have demonstrated that there are no placebo effects on pain sensation three and eight weeks after IAC injection. Nevertheless, further studies are necessary in order to conclude a causal association between the therapeutic effect of the injection and the improvements we found. The second limitation concerns the small sample size; in spite of that, we found significant differences between the evaluations before and after the treatment.

## 5. Conclusions

To sum up, following an intra-articular corticosteroid injection, relief of symptoms, improvement of spatiotemporal gait parameters, and increase of daily physical activity were observed. Although symptom relief and gait patterns are improved for at least six weeks, the effects on daily physical activity are very short-lived. These effects start to decrease from four weeks after injection and ultimately disappear after six weeks. Moreover, this study supports the claim that intra-articular therapeutic injections should be combined with biomechanical treatment to avoid excessive medial knee loading due to short-term symptomatic improvements. Further large-scale placebo-controlled studies are needed to confirm the beneficial effects of intra-articular corticosteroid injection on symptom relief and physical function in a real-life environment.

## Figures and Tables

**Table 1:** SF-36 scores (%) before and after injection: Means (SD).

SF-36 items	Before	2 weeks after	4 weeks after	6 weeks after	*P* value	Effect size	Observed power
Physical function	56.2 (25.1)	60.7 (21.8)	65.0 (21.3)	64.3 (22.3)	0.14	0.14	0.46
Physical limitation	54.0 (17.8)	65.4 (19.0)	67.8 (13.9)^†^	68.8 (14.0)^‡^	<0.01	0.29	0.87
Bodily pain	46.2 (19.1)	64.2 (15.3)^*^	68.1 (15.2)^†^	66.0 (13.2)^‡^	<0.001	0.44	0.99
General health	68.8 (11.8)	70.4 (12.2)	69.2 (13.2)	66.6 (12.0)	0.52	0.06	0.20
Energy/fatigue	59.6 (8.2)	63.8 (6.2)	66.2 (7.7)^†^	66.5 (8.3)^‡^	0.01	0.26	0.82
Social function	73.1 (20.3)	85.6 (16.0)^*^	89.4 (15.2)^†^	88.5 (10.8)^‡^	<0.01	0.31	0.91
Emotional limitation	84.0 (16.1)	85.3 (18.1)	82.7 (12.5)	84.0 (14.2)	0.83	0.01	0.08
Emotional well-being	73.5 (6.4)	71.7 (7.7)	73.8 (7.4)	72.6 (6.9)	0.63	0.05	0.16

^*^Significant difference between before and 2 weeks after injection.

^†^Significant difference between before and 4 weeks after injection.

^‡^Significant difference between before and 6 weeks after injection.

**Table 2:** Global and affected limb gait parameters before and after injection: Means (SD).

Parameter	Before	2 weeks after	4 weeks after	6 weeks after	*P* value	Effect size	Observed power
Velocity (m/s^−1^)	1.11 (.24)	1.21 (.17)^*^	1.20 (.19)^†^	1.21 (.20)^‡^	<0.01	0.29	0.87
Cycle time (s)	1.17 (.16)	1.12 (.15)	1.11 (.13)^†^	1.11 (.15)^‡^	<0.05	0.25	0.80
Cadence (step*·*min^−1^)	104 (14)	109 (13)^*^	110 (12)^†^	110 (13)^‡^	<0.05	0.28	0.85
Stride length (m)	1.28 (.17)	1.34 (.14)^*^	1.32 (.15)	1.32 (.14)	0.02	0.24	0.76
Walking base (cm)	9.8 (2.5)	8.9 (1.9)	9.3 (2.1)	9.0 (1.7)	0.13	0.14	0.47

^*^Significant difference between before and 2 weeks after injection.

^†^Significant difference between before and 4 weeks after injection.

^‡^Significant difference between before and 6 weeks after injection.
